# Towards Streamlined Transparent Data Linkage

**DOI:** 10.23889/ijpds.v9i5.2576

**Published:** 2024-09-18

**Authors:** Paul Kurdyak, Matthew Crocker, Anjie Huang, Natasha Saunders

**Affiliations:** 1 ICES; 2 Hospital for Sick Children

## Introduction

Most substance use disorder (SUD) treatment occurs in community settings. Community-based SUD treatment information is rarely captured or utilized. The objective of this study was to examine the efficiency of a data linkage of community-based SUD treatment to health administrative data holdings in Ontario, Canada, and to describe sociodemographic and clinical characteristics of individuals accessing SUD services.

## Methods

Data from community-based SUD service providers (>180) from April 2015 to March 2022 were linked to administrative data holdings at ICES. Linkage rates were evaluated. Sociodemographic (age, sex, neighbourhood-level income) and clinical (substance use, physician visits, Emergency Department (ED) visits and hospitalizations) characteristics were evaluated.

## Results

The linkage rate of DATIS ICES data holdings was >92% for all years (2015-2022). Of the 234,501 individuals admitted to a DATIS program, 36.1% were female, 46.2% were 25-44 years old, and 51.6% resided in the two lowest neighbourhood income quintiles. Alcohol (33.4%) was the most common substance identified. For outpatient care occurring within 1 year prior to admission, around 56.0% and 23.3% had Mental Health and Addictions (MHA) related primary care and psychiatrist visits, respectively. For acute health services, 29.8% and 14.2%  had a MHA- related ED visit or hospitalization, respectively.

## Discussion

SUD treatment data from community settings can be successfully linked to other health administrative data. Individuals with SUD have a high rate of acute health care use, and a relatively low access to psychiatrists. SUD treatment data linkage should be used to understand how to optimize access to care for vulnerable individuals.

